# Foreign Correspondence

**Published:** 1889-06

**Authors:** 


### Foreign Correspondence.

The following letter from the late lamented Dr. Brasseur has been handed us for publication. We were in possession of a promise from the Doctor of a communication on the subject-matter included in this letter, and which, except for his untimely demise, he would surely have furnished.—[Ed.]

To Dr. J. B. Davenport,
73 Bd Haussenaun, Paris.

My Dear Colleague:

You have had the kindness to read us, at a private meeting, a letter addressed to you by Dr. Thackston, in which he manifests to you his fear of seeing the French dentists take, for the Dental Congress that is to be held this year, the denomination of *International* Dental Congress.

Knowing and appreciating the high professional position of Dr. Thackston, I most earnestly desire to dissipate any doubts that may arise as to the object of our congress and I hasten to reply to his objections, even begging you, if necessary, to communicate to him my explanations.

Our colleague fears that if the French dentists give to the approaching congress the title of *International* Congress, and above all if this congress obtains all the success that it is destined to have, it will draw upon the entire profession the general dissatisfaction of the medicine body, and that consequently the dental section will be effaced from the programmes of future international medical congresses.

One fact has certainly contributed to this error of interpretation. Remember what happened in America when the International Medical Congress of Washington was formed. Dr. Kingsley made at that time the most strenuous efforts to separate the dental art from medicine and surgery, claiming that our art was special, having no connection with the medical art, and that dentists had no interest to take part in the International Congress. Is it not this recollection that to-day leads Dr. Thackston to communicate to you his fears, that we would be the first to entertain, if we had for one moment the idea of adopting Dr. Kingsleys views?

The proof of the contrary is clearly demonstrated by all our acts ever since we have been united and formed into a society, that is to say since 1879; to which society both you and your esteemed colleague Dr. Bogue belong, and of which, consequently, you can better than any others make known its strong medical attachment.

Did we not take an active part in the International Medical Congress of London, where for the first time our art formed a special section? Is not this also true of the congress at Washington, and are we not ready to participate in the one of Berlin in 1890? If we have taken the title of International Dental Congress it is because the congress has been formed with the support of the government and precisely on account of the great number of strangers of each profession that will be certain to visit the International Exhibition; this title has been imposed upon us as upon the sixty odd congresses of various societies that will meet at that epoch. We take advantage of this affluence of strangers in our Capital to convoke all dentists, whatever may be their nationality, and we consider this congress as absolutely independent of the International Medical Congress that is to meet at Berlin in 1890.

There will be in Paris during the exhibition, as I have said to you, a great number of congresses that will all be international, for instance, the Hygienic Congress, the Anthropological Congress, the Professional Medical Congress, etc., etc. We dentists, though having a special dental congress, can take part in these other congresses, because we may there find questions interesting us. Take for example the Professional Medical Congress (where will figure doctors, druggists, dentists and veterinary surgeons). These reasons which I have very sincerely given, are those that have caused us to accept the title of *International Congress* and which we are *absolutely obliged* to accept without modification.

I take this opportunity, my dear colleagues, to beg of you to make an earnest appeal in our name to the good will of our sympathetic American colleagues, asking them to come and take a large part in the congress. [Je profite de cette circonstance, mon cher confrére, pour vous prier de faire en notre nom un pressent appel a la bonne volonte de nos sympathiques confreres Américains pour qu'ils viennent prendre une large part a notre Congrés]. We know that in every place where there is question of science and profesional interest one is sure to find Americans with their practical ideas.

Receive, my dear colleague, the assurance of my best sentiments.

E. Brasseur,

President of the Société Odontologique,

Director of l'Ecole Dentaire de France.

Paris January, 15th 1889.

